# Advancing the concept of global oral health to strengthen actions for planetary health and One Health

**DOI:** 10.1186/s12939-024-02176-6

**Published:** 2024-04-15

**Authors:** Julian Fisher, Christian Splieth, Cleopatra Matanhire-Zihanzu, Michael Glick

**Affiliations:** 1 Center for Integrative Global Oral Health, University of Pennsylvania, School of Dental Medicine, 240 S. 40th Street, 3rd Floor East, Philadelphia, USA; 2 Präventive Zahnmedizin und Kinderzahnheilkunde, University of Greifswald, Fleischmannstr. 42, Greifswald, 17487 Germany; 3 Department of Oral Health, Faculty of Medicine and Health Sciences, University of Zimbabwe, MP167 Mt Pleasant, Harare, Zimbabwe

## Abstract

Advancing the concept of global oral health can help tackle the triple planetary crises of climate change, nature and biodiversity loss, and pollution and waste. A model for oral and planetary health places more explicit focus on understanding the state of the Earth’s systems, changing environment in relation to planetary health boundaries and their impact on human well-being. This can facilitate a planet-centric critical thinking for equity in global oral health that contributes to UN 2030 Agenda for Sustainable Development.

The World Health Organization (WHO) Global Strategy on oral health and its complementary Global Oral Health Action Plan 2023 – 2030 come at a critical juncture for the health of the planet with recent international scientific reports and conferences signaling the urgent need for action [1, 2]. Many governments and societies around the world are already experiencing the impact of climate change and the risks associated with moving beyond the safe operating space for humanity and the planet [3]. The World Ecomonic Forum Global Risk Report 2024 emphasizes that no one is or will be safe from climate change risks and highlighted a predominantly negative outlook for the world over the next two years that is expected to worsen over the next decade [4]. Biodiversity loss, ecosystem collapse, and critical change to Earth systems will be felt most acutely by the most vulnerable populations [5]. These concepts are mediated through individuals’ material circumstances and compounded by the lack of necessary capital to protect themselves and their families against rising economic instabiity and shocks due to climate change.

Climate change is identifed as one of the nine planetary health boundaries and is increasing its negative impacts on society, including on health systems and the health of individuals [6]. Climate-sensitive health risks, their exposure pathways and vulnerability factors will place additional stresses on the social determinants of oral health, such as livelihoods, education, housing, as well as on the oral health care services in the general health and social system [7].

Climate change is a threat multiplier. It will slow and potentially reverse progress on achieving essential oral health services as part of Universal Health Coverage, as well as compound the morbidity and mortality due to oral diseases and conditions, thereby adding to an already urgent public health challenge. Wide-spread flooding, severe and prolonged heat waves and forest fires causing air pollution, all due to the impact of climate change, will exacerbate existing barriers to accessing oral health services, often when and where disadvantaged population groups need them most. Climate change poses a significant threat to traditional indigenous medical practices in communities with long-standing cultural and beneficial knowledge, skills and practices where herbal medicines are used to improve oral health.

The growing threat to the health of the planet and as a consequence a threat to human health, are complex, trans-boundary, multi-level and sector wide. This challenges us to adopt planet-centric critical thinking. Health and oral health gains will need to be considered in relation to their full cost to the Earth’s natural systems and not only related to the degradation of the environment (from hazardous chemicals), supply chain activities (material sourcing, manufacturing, and distribution) and ineffective waste management and disposal.

The concept of planetary health is based on the understanding that a healthy environment -the conditions in which people are born, grow, work, live, and age - is needed to support healthy communities [8]. Planetary health addresses the forces and systems – political, economic, and social – that shape the conditions of daily life and the health of the Earth’s natural systems [9]. It is important to recognize that planetary health and One Health are not mutually exclusive to other frameworks, which all share the common idea of planet Earth and its ecosystems as our home. We have the political consensus and analytical tools to tackle these complex problems; measure and understand, act and assess the impact of action. Safeguarding the Earth’s natural systems are the foundation of the UN 2030 Agenda for Sustainable Development and its 17 Sustainable Development Goals (SDGs) [10]. These goals and their 169 targets will stimulate action in five areas of critical importance for humanity and the planet; people, planet, prosperity, peace and partnerships. Such an action-oriented framing can encourage both long-term considerations for sustainable development, as well as short and mid-term political and organisational decision-making and reporting for sustainability (Fig. 1).

**Fig. 1:**
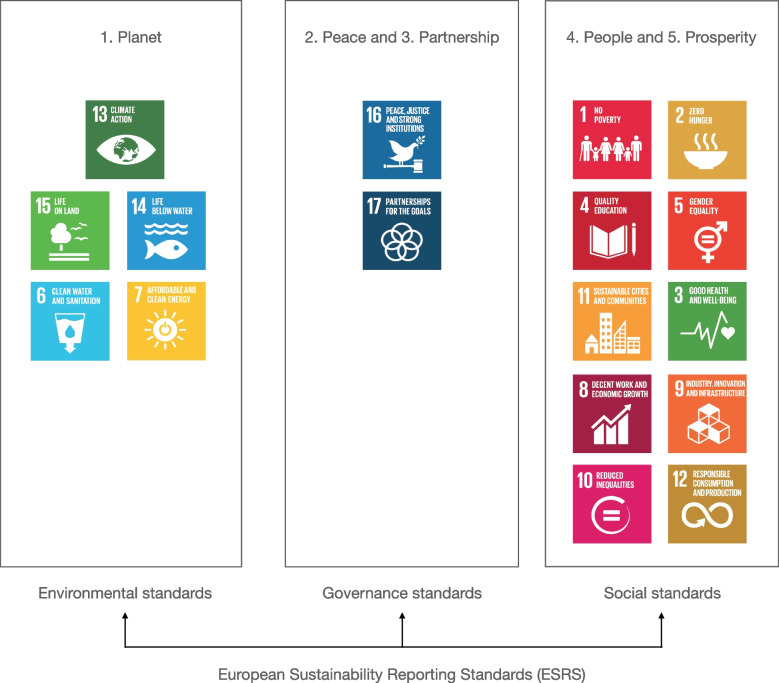
Action-oriented framing for both long-term considerations for sustainable development and short and mid-term political and organisational decision-making and reporting for sustainability

Recent WHO and FDI World Dental Federation definitions of oral health are transformational and can act as the calayst for a progressive global oral health agenda [11, 12]. These definitions create a narrative for sustainable oral health that enables a shift from a bio-medical model of care to one that recognizes the full spectrum of health and oral health, where oral health is understood to be influenced by a wide range of bio-psychosocial-spiritual perspectives and the healthy functioning and integrity of the Earth’s systems [13].

Visual models for sustainable development, such as the doughnut concept, set out ecological aspects as ceiling for survival and the social aspects as foundation for human world [14]. This concept describes a doughnut-shaped space or a corridor between ecological planetary boundaries and social foundations that is both ecologically safe and socially just: a space in which humanity can thrive. A model for oral and planetary health places more explicit focus on understanding the state of the Earth’s systems, changing environment in relation to planetary health boundaries and their impact on human well-being. The aim is to promote planet-centric critical thinking for equity in global oral health that contributes to UN 2030 Agenda for Sustainable Development (Fig. 2).

**Fig. 2:**
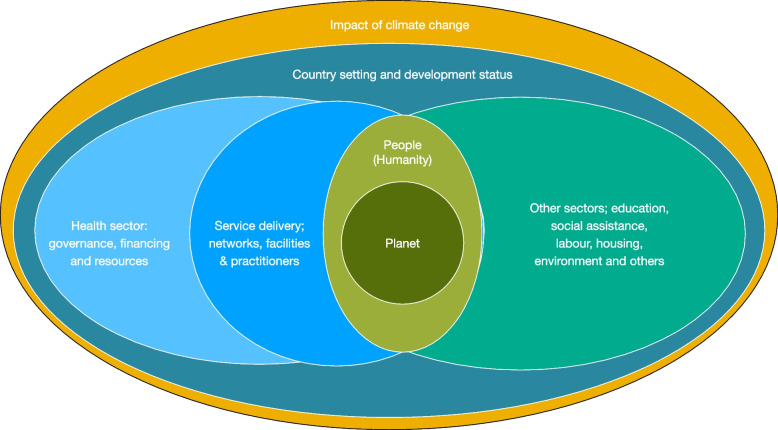
A model for oral and planetary health adapted from WHO Framework on Integrated People-Centred Health Services

New thinking around oral and planetary health can advance the definition of global oral health in two ways [15]. Firstly by promoting sustainable oral health where partnerships within and outside the oral health sector deliver services and provide care that meet the needs of today’s populations without compromising the ability of health systems to meet the needs of future generations - a transdisciplinary approach. Secondly, that people and planet-centered approaches should catalyze and empower inclusive, collective and long-lasting partnerships, that enable people to maintain their oral health in daily life and in the face of adversity, trauma, threat or significant sources of stress throughout their lifespan.

Sustainable oral health acknowledges that the power, wealth, and distribution of resources hierarchies, which significantly impact the access and utilization of Earth's natural resources, lead to environmental and social inequalities within communities. These disparities reinforce historical legacies and ongoing injustices, directly and indirectly affecting oral health outcomes in the short, medium, and long term.

A continuously evolving transdisciplinary model together with a planet-centric approach to global oral health are necessary to respond to the world’s changing political, social, economic and environmental circumstances. In doing so, efforts towards improving both oral health and planetary health can be synergized. This would align well with the WHO Global Oral Health Action Plan. The WHO Action Plan promotes the planetary health-appropriate use of natural resources, such as water and energy, as well as calling for safe and environmentally sound oral health supplies and consumables and oral care products. A central tenet of the WHO Action Plan is the adoption of environmentally friendly, less invasive dentistry and use of essential medicines. This contributes significantly to developing climate-resilient and low-carbon oral health systems, as well as enhancing disaster preparedness and response [16]. It ensures that care can be sustained by utilizing essential materials and techniques when traditional restorative treatments are impractical or not the optimal approach.

This enhanced concept of global oral health resonates with the WHO publication ‘Integrating the social determinants of health into health workforce education and training’ and its chapters on oral health, and food security, food safety and nutrition [17]. Today’s learners who will become the global oral health leaders of the future must receive education and training on the social determinants of health, which is an important part of the larger agenda to transform health workforce education for sustainable development, as recommended by the report of the United Nations High-Level Commission on Health Employment and Economic Growth [18]. New ways of educating and training for oral health can highlight entry points for advocacy in political forums outside the health sector but which impact health and oral health. For example concern about the fragility of the world’s food systems and their interconnectedness with other systems such as economic, geopolitical, health, energy and ecosystems. These complexities and the impact of climate change will make the global food system more vulnerable to crisis and supply chain breakdown, which links food security to health security [19].

A model for oral and planetary health reflects the evolution and expansion of the One Health approach to promote sustainable and resilient health and well-being [20]. It recognizes the health of people is closely connected to the health of animals and our shared environment and offers promising solutions for addressing unprecedented challenges. One Health is an approach to designing and implementing programmes, policies, legislation and research in which multiple sectors communicate and work together to achieve better public health outcomes. The fourth High-level Meeting of the United Nations General Assembly in 2025 offers a strategic opportunity for global oral health advocates and champions to support integrated systems and strengthening capacity to address complex multidimensional health risks. This can create additional avenues and platforms to contribute decision-making on a set of 9 voluntary global targets for the prevention and control of noncommunicable diseases including oral diseases [21].

In conclusion, advancing the concept of global oral health can help tackle the triple planetary crisis of climate change, nature and biodiversity loss, and pollution and waste. This concept can facilitate a more comprehensive approach that fosters transdisciplinary, multi-level, and cross-sector collaboration and make an active contribute to a future we want [22].

## Data Availability

No datasets were generated or analysed during the current study.
